# Supplementary material for: Quantum capacitance mediated carbon nanotube optomechanics
Source: Nat Commun. 2020 Apr 2;11:1636. doi: 10.1038/s41467-020-15433-3 (PMC7118114; doi:10.1038/s41467-020-15433-3)
Supplement: Supplementary file 1 — Supplementary Information [file 41467_2020_15433_MOESM1_ESM.pdf]

Supplementary Information

Quantum capacitance mediated carbon nanotube optomechanics

S. Blien *et al.*

Supplementary Note 1 — Coplanar resonator fabrication

Fabrication starts with a substrate of 500 μm compensation doped silicon, covered by 500 nm thermally grown SiO_2 and 100 nm niobium. The niobium layer is structured to form the coplanar $\lambda/2$ resonator geometry (1) (see Supplementary Figure 1) using optical lithography and reactive ion etching with argon and sulphur hexafluoride SF_6. In this step also the bond pads (2) (3) (5) and the outer dc leads are defined, though the dc leads are not connected to the nanotube deposition area and the resonator center line yet.

Next, using electron beam lithography and thermal deposition of gold, the 100 nm wide and 50 nm thick gate finger (8) is created near the coupling capacitors of the resonator, reaching under the area where a carbon nanotube will be deposited. This gate finger is then covered with about 200 nm of poly-methyl methacrylate (PMMA) electron beam resist, which is locally cross-linked via electron beam overexposure. The subsequent step uses the same techniques as for the gate finger to connect the gate dc lead with the centre of the resonator, and to create the source, drain, and cutting electrodes for the nanotube (6) using 200 nm gold.

As final step, areas to either side of the transfer electrodes are etched 10 μm deep into the substrate (7), to allow the nanotube transfer fork (see below) to pass below the resonator plane.

Supplementary Note 2 — Carbon nanotube growth and deposition

Our carbon nanotube growth and deposition process is based on techniques developed by several research groups over the past years; a more detailed description can be found in Ref. 1. As growth substrate, we use a commercial, macroscopic quartz tuning fork, where the metallization has been chemically removed. Nominally 1 nm cobalt is sputter-deposited onto the fork tips. Subsequently, the quartz fork is inserted into a tube oven and exposed to a gas flow of 10 sccm CH_4 and 20 sccm H_2 at 960°C for 30 min. The cobalt clusters on the tip surfaces then act as catalyst seeds for the nanotube growth.[2,3]

A typical growth result is shown in the scanning electron micrograph of Supplementary Figure 2a, where long and clean carbon nanotubes cross the fork tines and the gap between them. Here, for better visibility of the nanotubes, the entire fork has been coated with cobalt, not only its tips.

Subsequently, the fork is mounted on a manual positioning system and lowered towards the sample holder circuit board with the bonded resonator chip, see Supplementary Figure 2b. Monitoring the current between the contact electrodes allows us to detect when a nanotube touches them. Then, between each of the outer two electrode pairs, a voltage is subsequently ramped up to cut the macromolecule with a high current. After cutting, the fork is retracted, with a nanotube segment remaining between the central source and drain electrodes.

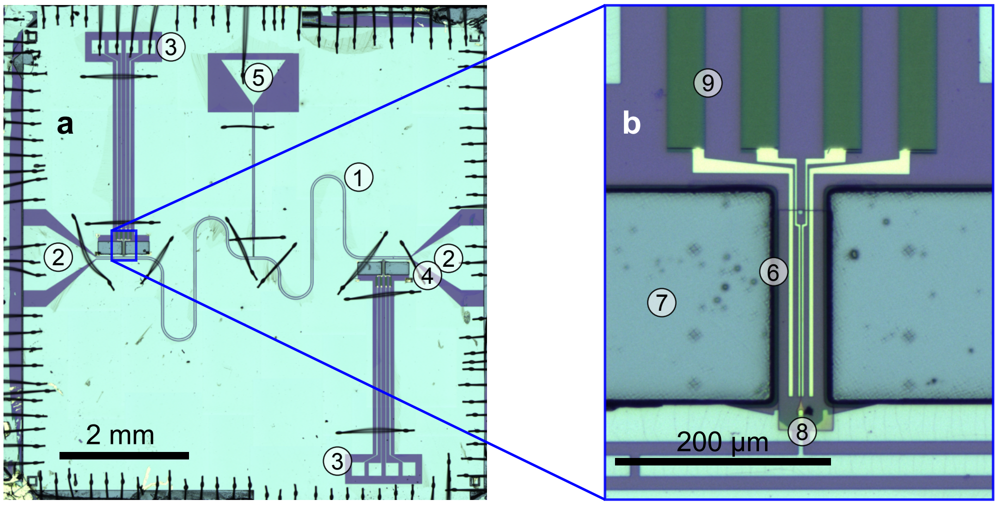

Supplementary Figure 1. **The combined coplanar waveguide resonator – carbon nanotube device** **a**, Optical microscope overview image, including the superconducting coplanar resonator (1) (length $\sim$ 1 cm), the bond pads (2) to couple a GHz signal into and out of the cavity, the dc bond pads (3) leading to the transfer areas (4), and the dc bondpad (5) leading to the center conductor of the cavity. **b**, Detail zoom of the carbon nanotube deposition area with the transfer electrodes (6) in between the deep etched areas (7), the gate finger (8) connected to the cavity, and 0.5 μm thin, meandering gold lines (9) as rf blocks, connecting the dc electrodes to the bondpads shown in **a**.

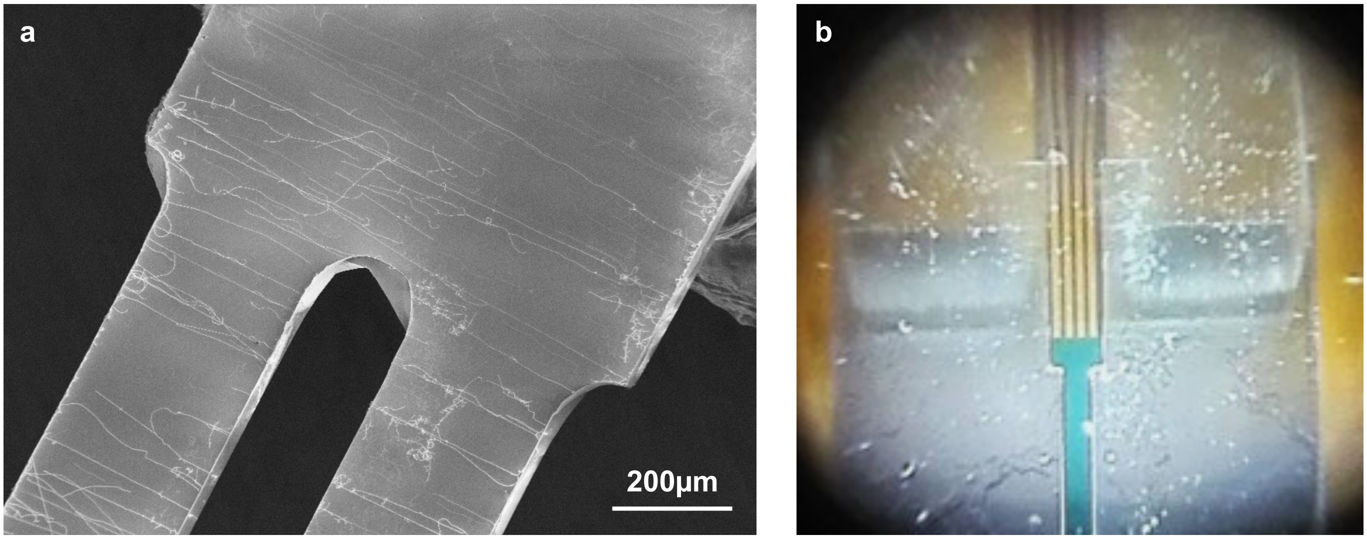

Supplementary Figure 2. **Nanotube growth and transfer** **a**, Scanning electron micrograph of carbon nanotubes grown over and between the tines of a commercial quartz tuning fork[1]. For better visibility of the nanotubes, here the entire fork has been covered with cobalt catalyst, not only the fork tips. **b**, Optical microscope view during nanotube transfer. A near-transparent growth quartz fork is lowered onto an electrode chip (here without a coplanar resonator, for testing purposes), while monitoring the resistance between the target electrodes.

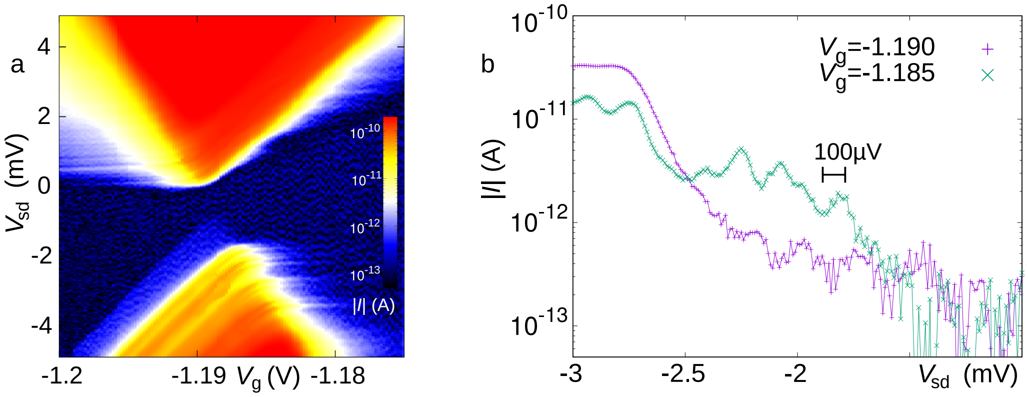

Supplementary Figure 3. **Characterization of the Coulomb blockade oscillation** **a**, Logarithmic color scale plot of the absolute value of the dc current $|I(V_g, V_{sd})|$ as function of gate voltage V_g and bias voltage V_{sd}, in the parameter region discussed in the main manuscript. A small current offset, likely caused by the used current-to-voltage amplifier, has been manually corrected. Note that over the duration of the measurement the absolute gate voltages of the Coulomb oscillations underwent a significant slow shift. **b**, Trace cuts from a, with a characteristic bias voltage scale of 0.1 mV indicated.

Supplementary Note 3 — Quantum dot precharacterization

Supplementary Figure 3a displays a detail measurement of the Coulomb oscillation of the nanotube near $V_g = -1.2\,\mathrm{V}$. A gap in low-bias conduction across Coulomb diamonds, as visible here and also in Figure 1c of the main text, can be caused by a chain of several quantum dots.[4,5] The regularity of the diamond-shaped regions of Coulomb blockade in Figure 1c, where overall only one set of delineating slopes occurs, indicates that one quantum dot is dominant; if a second potential minimum exists, it is likely a trap state at one of the contacts, e.g., pinned to that contact's potential.

In order to obtain a lower estimate of the total tunnel rate Γ into and out of the quantum dot, we look at the sharpest current features in single electron tunneling at this Coulomb oscillation, see Supplementary Figure 3b. The typical scale is given by $\Delta V_{sd} = 0.1\,\mathrm{mV}$, corresponding to a characteristic energy of $\Delta\varepsilon = 0.064\,\mathrm{meV}$ and a rate $\Gamma_{est} = \Delta\varepsilon/h \simeq 15\,\mathrm{GHz}$. This still exceeds the cavity resonance frequency of $\omega_c/(2\pi) \simeq 5.74\,\mathrm{GHz}$, making separation of timescales a valid assumption.

We note here however that the current as function of bias voltage exhibits peaks, not steps as expected for a single quantum dot, within the single electron tunneling and co-tunneling regions. This is an additional indication for a trap state and quasi

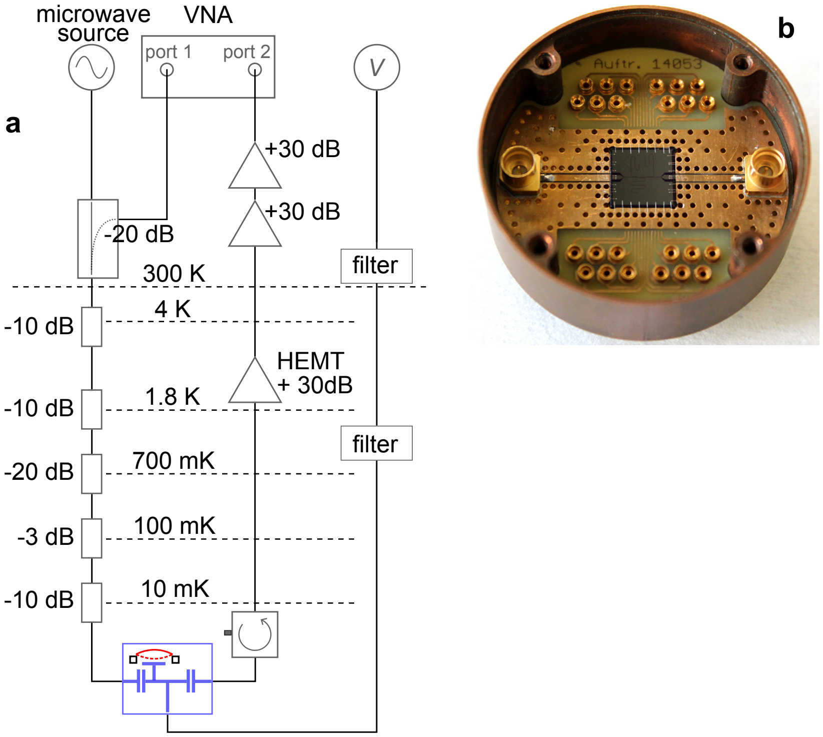

Supplementary Figure 4. **Cryogenic measurement setup** **a**, Schematic measurement setup, at the example of an optomechanically induced transparency (OMIT) measurement. **b**, Top view of the sample holder with dc and GHz access to the chip. The dc lines, with 12 vias at upper and lower edge of the PCB each, lead to a connector on the bottom side.

double quantum dot behaviour, where discrete levels tune into and out of resonance; in such a system the intrinsic line width of a weakly coupled trap level may additionally cause feature sizes.

A near-zero bias trace of the dc current, see Supplementary Figure 12, is consistent with a broadening $\Gamma \sim 163\,\mathrm{GHz}$.

Supplementary Note 4 — RF measurement setup

Supplementary Figure 4a sketches the measurement setup integrating both dc and GHz access into our Oxford Instruments Kelvinox 400HA dilution refrigerator; the arrangement of the room temperature equipment in the figure corresponds to the optomechanically induced transparency (OMIT) measurement shown in Figure 2 of the main text. The probe signal, generated by a Rohde & Schwarz (R&S) ZVA 24 vector network analyzer, is injected into the coupled port, the drive signal, generated by a R&S SMB100A microwave source, into the direct port of a directional coupler at room temperature. UT85 semirigid cables, using superconducting NbTi from the 1K stage on, and with thermally anchored attenuators inserted at every temperature stage, convey the combined signal to the device at dilution refrigerator base temperature.

Our chip is mounted on a custom-made printed circuit board “sandwich”, with two Mini-SMP connectors for the GHz signals and a Micro-D connector for up to 24 dc wires, see Supplementary Figure 4b. A cryogenic circulator, mounted at the mixing chamber stage, with a terminated third port, guides the transmitted output signal towards the cryostat output and blocks noise from higher temperature stages. The signal is amplified by 30 dB each along a chain of amplifiers: a cryogenic low-noise HEMT amplifier Caltech CITCRYO 1-12 at the 1K-pot stage, and two microwave amplifiers at room temperature. Subsequently, it is detected by the vector network analyzer.

Supplementary Figure 5, extending Figures 1b and 2b of the main text, combines a simplified sketch of the device and a circuit model to clarify device function and nomenclature.

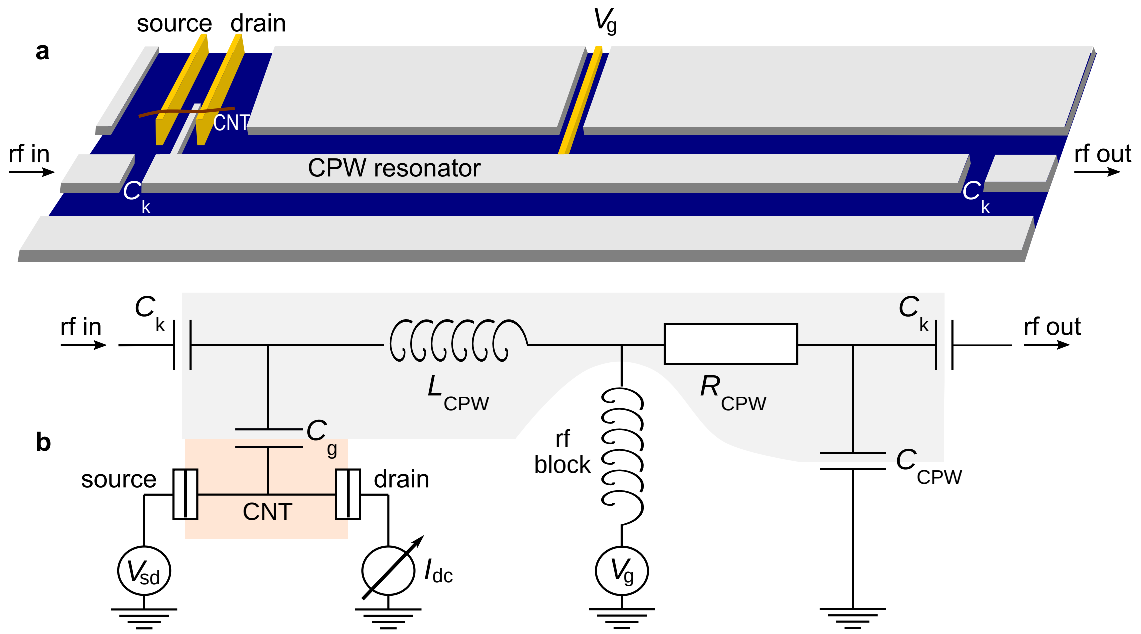

Supplementary Figure 5. **Models of the optomechanical device** **a**, Schematic drawing and **b**, corresponding circuit. The carbon nanotube, marked in brown in the model, is capacitively (C_g) coupled to a coplanar waveguide resonator (R_{CPW}, L_{CPW}, C_{CPW}), marked in gray in the model. A microwave signal can be coupled in and out of the cavity via coupling capacitors (C_k). To tune the electrostatic potential, an additional dc gate voltage V_g can be applied to the cavity center conductor.

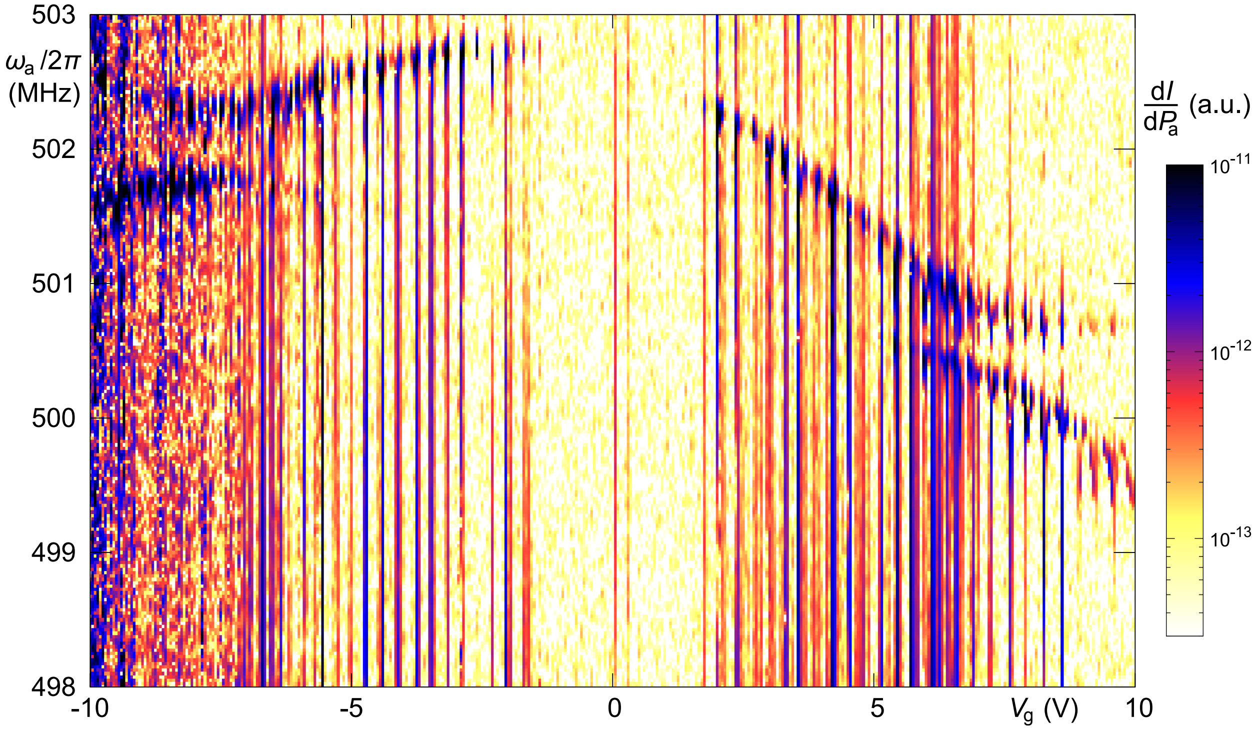

Supplementary Figure 6. **Carbon nanotube vibration mode precharacterization, raw data** Absolute value of the lock-in signal $|dI/dP_a|$ measured while an amplitude-modulated rf signal is irradiated onto the device, as function of signal frequency $\omega_a/2\pi$ and applied gate voltage V_g. Tracewise extraction of the peak positions leads to the data plotted in the main text, Figure 1d, and in Supplementary Figure 7.

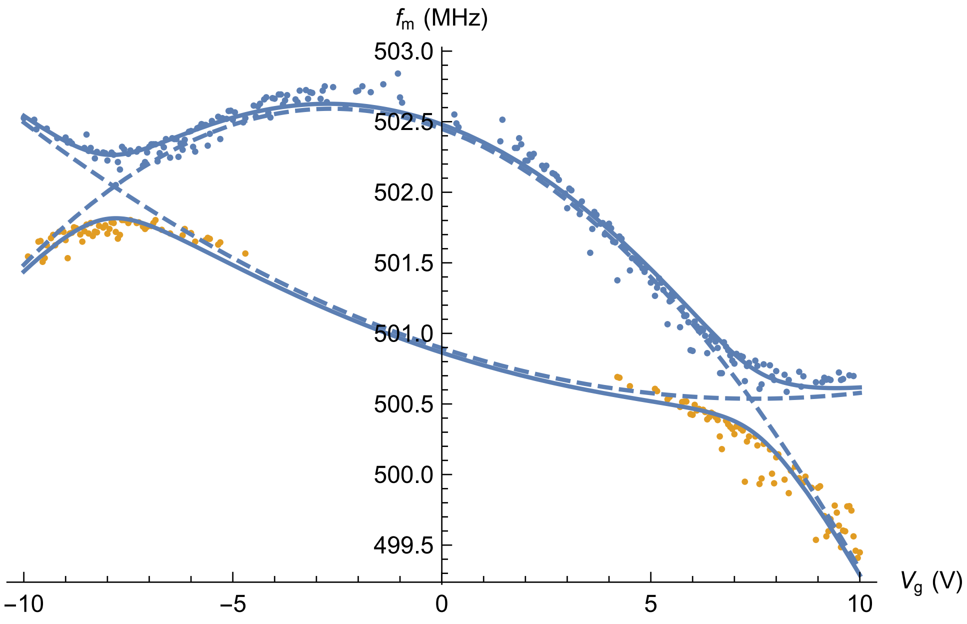

Supplementary Figure 7. Carbon nanotube vibration mode precharacterization Resonance positions extracted from the data of Supplementary Figure 6, and fitted using two coupled modes with parabolic dispersion each.

Supplementary Note 5 — Vibration mode precharacterization

In order to precharacterize the vibration modes of our carbon nanotube device, following Refs. 6–9 we use an antenna to irradiate an amplitude-modulated MHz signal onto the device and measure the current response through the device using a lock-in amplifier. The result is plotted in Supplementary Figure 6; two gate-dependent vibration modes are clearly visible, which display an avoided crossing at $V_g \simeq \pm 8\,\mathrm{V}$. A trace-wise extraction of the response maxima leads to the plot of Figure 1d in the main text. Even though the contact separation of our device is $L = 1\,\mu\mathrm{m}$, we do not observe any vibration modes at lower frequency. In combination with the very weak gate voltage dependence of the resonance frequencies, this indicates that high mechanical tension has been imprinted on the carbon nanotube during device fabrication.

In Supplementary Figure 7, the two observed modes are fitted regarding their gate voltage dependence. We use for the bare mode frequency in both cases as approximation a quadratic dispersion, in one case with positive, in the other with negative curvature. In addition, a single mode coupling parameter common to both observed anticrossings is introduced.

Both dispersions with positive and with negative curvature have been observed in literature; the former corresponds to gate voltage-induced tension,[6,10] the latter to electrostatic softening of the vibration.[11,12] The frequency maximum of the softening mode is near the apparent band gap as expected. The tension-dominated mode has a frequency minimum near $V_g = 7\,\mathrm{V}$, but also only a very small curvature. We cannot give an explanation for this phenomenon.

For both observed resonances, the obvious gate voltage dependence of the resonance frequency excludes an electromagnetic resonance in the measurement apparatus as origin. Additionally, both modes show in dc current measurements the characteristic oscillatory vibrational softening at Coulomb blockade oscillations,[7] see Supplementary Figure 8.

Supplementary Note 6 — Cavity precharacterization

For a $\lambda/2$-resonator, as the one used here, intrinsically a Lorentzian line shape of the transmission as function of frequency is expected. As also frequently observed in literature, the measured signal can, however, deviate, due to crosstalk on the sample around the actual resonator. The combination of a resonant channel and direct transmission leads to a Fano line shape, see Supplementary Figure 9, where $|S_{21}|^2$ is plotted both as linear value and in dB.

In a fit model, the side channel corresponding to the crosstalk is introduced by adding an additional complex term[13] $e^{i\theta} r$,

$$S_{21} = A\left[e^{i\theta} r + \frac{1}{\frac{\kappa_c}{2} - i(\omega - \omega_c)}\right]. \tag{1}$$

Here, A is an overall transmission amplitude, and ω_c and κ_c are the cavity resonance frequency and line width. The fit function

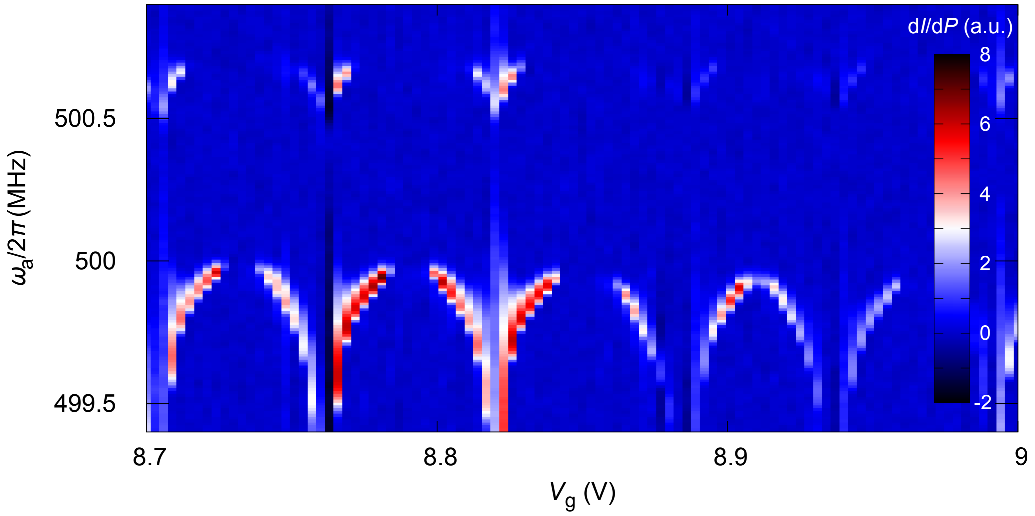

Supplementary Figure 8. **Carbon nanotube vibration mode precharacterization, detail** "Coulomb blockade oscillations of vibrational resonance frequency", cf. Ref. 7, of both observed resonances.

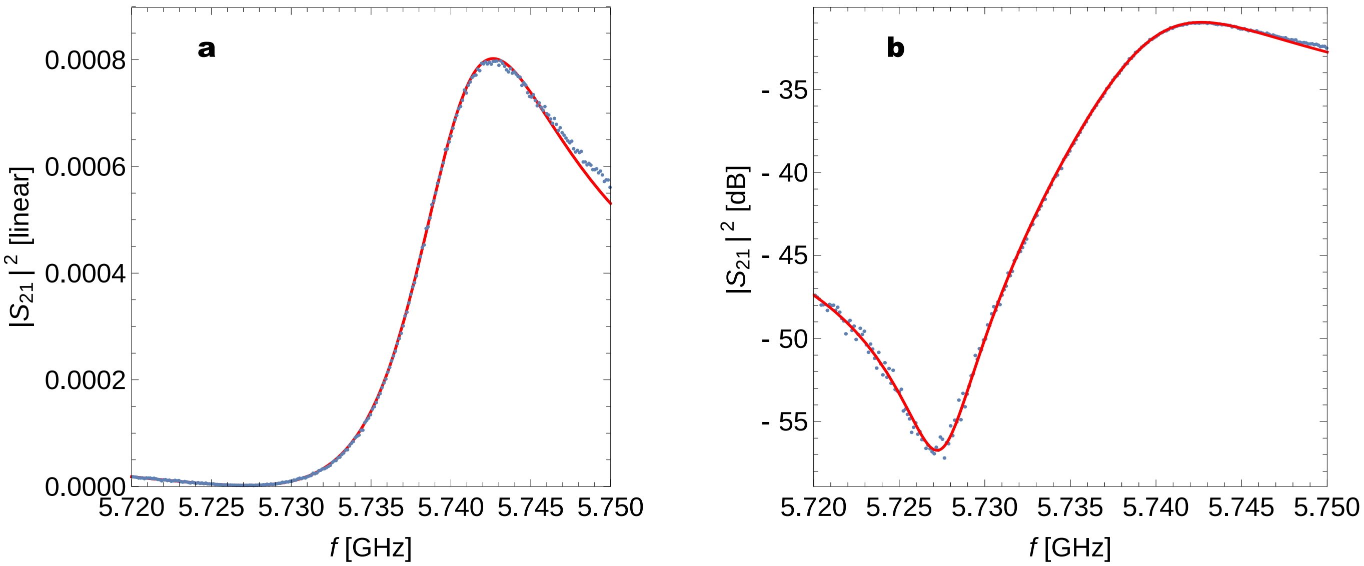

Supplementary Figure 9. **Characterization of the coplanar waveguide cavity transmission near its resonance** **a**, power transmission in linear scale, **b**, power transmission in dB. Points are measured transmission data; the solid line is a fit of Supplementary Equation 1.

of Figure 9 corresponds to $\omega_c/2\pi = 5.74005\,\text{GHz}$ and $\kappa_c/2\pi = 11.6\,\text{MHz}$, leading to a total quality factor of $Q_c = 497$. The fit parameters for the crosstalk side channel are $\theta = -39.5791^\circ$ and $r = -7.53961 \times 10^{-8}\,\text{s}$.

Supplementary Note 7 — Cavity photon number calibration

Since the center conductor of the coplanar microwave cavity also acts as dc gate of the carbon nanotube quantum dot, we use the cavity-induced broadening of Coulomb oscillations for calibration of the voltage amplitude in the cavity and thereby the average photon number. Modeling the microwave cavity classically, it applies a sinusoidal ac gate voltage on top of the dc

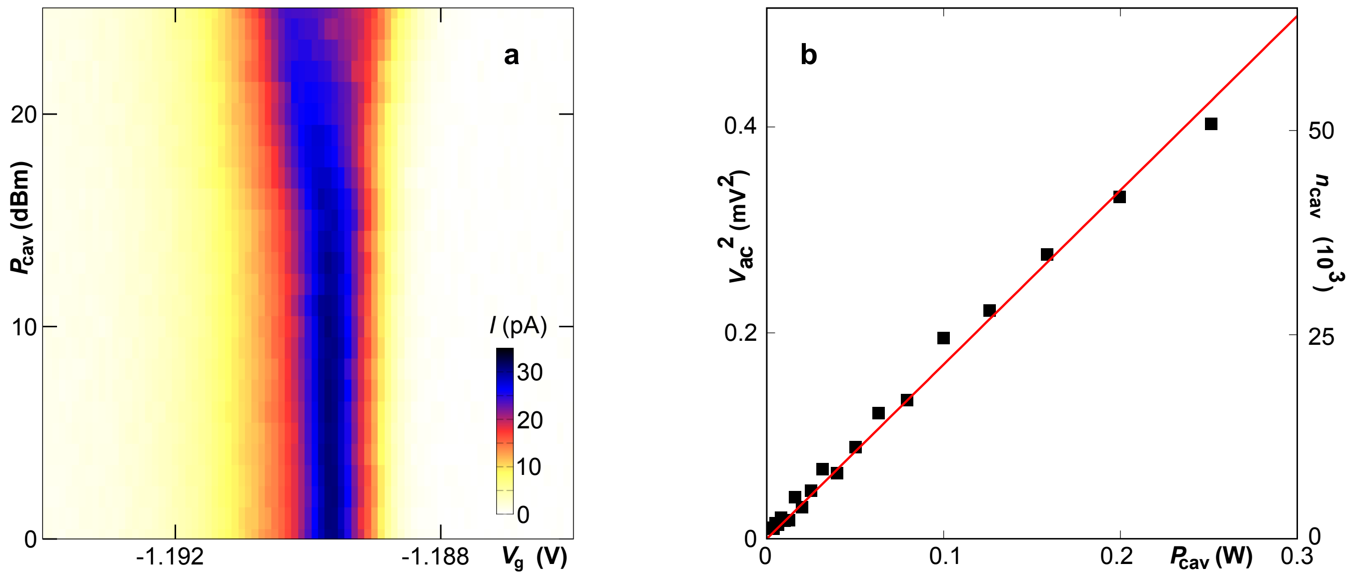

Supplementary Figure 10. **Cavity photon number calibration** **a**, Current across a Coulomb blockade oscillation, as a function of gate voltage V_g and off-resonant, red sideband ($\omega_d = 2\pi \cdot 5.2399\,\text{GHz}$) cavity drive power P_{cav} (as set at the signal generator). For increasing drive power, the current peak broadens. **b**, Extracted squared ac gate voltage amplitude $(V_g^{ac})^2$ (left axis) and cavity photon number n_c (right axis), as function of applied generator drive power P_{cav}.

voltage. In a dc measurement, this leads to broadening of a Coulomb oscillation $I(V_g)$ as follows:

$$I_{\text{driven}}(V_g, P_{cav}) = \frac{1}{2\pi} \int_0^{2\pi} I\left(V_g + V_g^{ac}(P_{cav})\sin(\varphi)\right) \mathrm{d}\varphi \tag{2}$$

where $V_g^{ac}(P_{cav})$ is the so-far unknown dependence of the ac voltage amplitude on the drive power. Note that both voltages V_g and V_g^{ac} are applied via the same gate electrode and thus act on the quantum dot with the same geometric lever arm.

The function $I(V_g)$ is extracted for the lowermost generator power, $I(V_g) := I_{\text{driven}}(V_g, -10\,\text{dB})$, and Supplementary Equation 2 is used to obtain $V_g^{ac}(P_{cav})$ by fitting $I_{\text{driven}}(V_g, P_{cav})$, Supplementary Equation 2, for each power setting P_{cav} at the generator. The result is shown in Supplementary Figure 10b (left axis).

For obtaining the number of photons in the cavity n_c, we calculate the total capacitance of our coplanar waveguide based on conformal mapping techniques;[14] this results in $C_{\text{CPW}} = 1.75\,\text{pF}$. The energy stored in the cavity is then given by

$$E = \frac{1}{2} C_{\text{CPW}} \left(\frac{V_g^{ac}}{\sqrt{2}}\right)^2 = n_c \hbar \omega_d, \tag{3}$$

where the factor $1/\sqrt{2}$ reflects the non-homogeneous voltage distribution along the cavity.[15] The resulting cavity photon number n_c is given by the right axis of Supplementary Figure 10b, and depends, as expected, linearly on the drive power. For a generator output power of $P_{cav} = 25\,\text{dBm}$, we obtain $n_c = 67501$.

Supplementary Note 8 — Optomechanical coupling

A key parameter of a dispersively coupled optomechanical system[16] is the single-photon optomechanical coupling g_0, given by the shift in cavity frequency when the mechanical resonator is displaced on its characteristic zero point fluctuation length scale x_{zpf},

$$g_0 = \left.\frac{\partial \omega_c}{\partial x}\right|_{x=0} x_{\text{zpf}}. \tag{4}$$

In experiments, it typically enters via

$$g = g_0\sqrt{n_\mathrm{c}}, \tag{5}$$

where g is the optomechanical coupling scaling with the number of cavity photons n_c.

To derive g_0 for our coplanar waveguide cavity, we start with the electronic cavity parameters. The cavity can be modeled as a lumped element resonator with total capacitance C_c, approximated as a sum of three terms,

$$C_\mathrm{c} = \frac{C_\mathrm{CPW}}{2} + 2C_\mathrm{k} + C_\mathrm{g}(x). \tag{6}$$

The capacitance C_CPW of the coplanar waveguide forming the cavity to its ground plane can be calculated analytically from the waveguide geometry, using conformal mapping techniques.[14,15] For our device we obtain $C_\mathrm{CPW} = 1.75\,\mathrm{pF}$; the factor 1/2 stems from the transformation between distributed and lumped element model.[15] C_k describes the coupling of input (drive) and output line to the cavity; it can be estimated by finite element modelling, is typically in the range of fF,[15] and in any case small for an undercoupled cavity. The last and by far smallest term, $C_\mathrm{g}(x)$, is the deflection-dependent capacitance between the suspended carbon nanotube and the cavity gate finger; thus, to a good approximation

$$C_\mathrm{c} \simeq \frac{C_\mathrm{CPW}}{2} \tag{7}$$

With $\omega_\mathrm{c} = 1/\sqrt{C_\mathrm{c}L_\mathrm{c}}$, the optomechanical single-photon coupling strength g_0 from Supplementary Equation 4 is then given by[16,17]

$$g_0 = \frac{\omega_\mathrm{c}}{2C_\mathrm{c}} \left.\frac{\partial C_\mathrm{c}}{\partial x}\right|_{x=0} x_\mathrm{zpf}. \tag{8}$$

Supplementary Note 9 — Extracting the coupling constant from OMIT

Since we measure transmission *through* our $\lambda/2$ cavity, from an input port to an output port, we need to take both input and output coupling separately into account. We define the loss rate of the GHz cavity κ_c by

$$\kappa_\mathrm{c} = \kappa_0 + \kappa_\mathrm{in} + \kappa_\mathrm{out} \tag{9}$$

where κ_in describes the input port and κ_out the output port coupling, and κ_0 is the intrinsic loss. Following Ref. 18, in an OMIT experiment, with s_in as amplitude of the *probe* signal at ω_p, with a cavity *drive* signal at the red-detuned mechanical side band $\omega_\mathrm{d} = \omega_\mathrm{c} - \omega_\mathrm{m}$, and with $\Delta = \omega_\mathrm{p} - (\omega_\mathrm{d} + \omega_\mathrm{m})$, the intracavity *probe* field amplitude is given by

$$A(\Delta, g) = \frac{1}{(-i\Delta + \kappa_\mathrm{c}/2) + \frac{g^2}{-i\Delta + \kappa_\mathrm{m}/2}} \sqrt{\kappa_\mathrm{in}} s_\mathrm{in}. \tag{10}$$

leading to a signal transmitted through the cavity

$$s_\mathrm{out}(\Delta) = \sqrt{\kappa_\mathrm{out}} A(\Delta, g) \tag{11}$$

We need to consider again the crosstalk transmission that leads to the Fano cavity line shape; it bypasses the optomechanical coupling as well and is thus not influenced by OMIT. To model it, as in Supplementary Equation 1, we add a complex shift

$$A_\mathrm{Fano}(\Delta, g) = \left(e^{i\theta} r + \frac{1}{(-i\Delta' + \kappa_\mathrm{c}/2) + \frac{g^2}{-i\Delta + \kappa_\mathrm{m}/2}} \right) \sqrt{\kappa_\mathrm{in}} s_\mathrm{in}. \tag{12}$$

The parameters r and θ, which characterize our setup (e.g., the sample holder), are taken from the fit of Supplementary Note **6** and kept constant. Additionally, following Ref. 18, we account for drive frequencies ω_d that deviate from the motional sideband condition $\omega_\mathrm{c} - \omega_\mathrm{m} = \omega_\mathrm{d}$ by a modified parameter $\Delta' = \omega_\mathrm{p} - \omega_\mathrm{c}$.

With this, the OMIT data can be fitted; the power transmission coefficient $|S_{21}|^2$ recorded by the vector network analyzer is proportional to $|A_\mathrm{Fano}(\Delta, g)|^2$. Supplementary Figure **11** illustrates the result at the example of the data set of Figure 2c of the main text. Note that in Figure 2c a strong probe signal was applied, leading to a nonlinear response of the mechanical system,[19] which is why the fit using a linear response function here only provides a rough approximation. For the data of Figure 2f, the signal strength has been reduced to obtain a linear response.

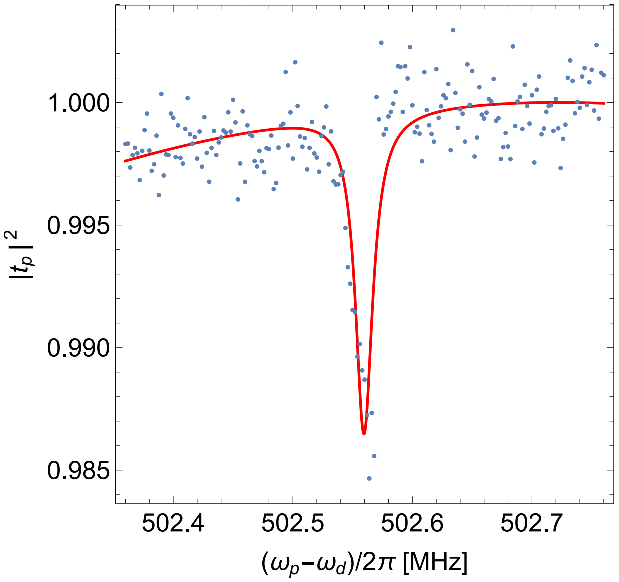

Supplementary Figure 11. **OMIT data evaluation** Normalized power transmission of the data trace of Figure 2c in the main text, with a corresponding fit based on Supplementary Equation 12. Note that here the applied probe power is large, leading to a nonlinear response of the mechanical system; the fit can only be seen as a rough approximation.

Supplementary Note 10 — Regarding sign conventions

When mathematically treating systems of one or multiple oscillating observables with the help of the exponential function with imaginary argument, different sign conventions are possible. This becomes immediately visible when comparing the ansatz of Ref. 18, the first OMIT observation, with, e.g., Ref. 20, a standard reference for microwave-optomechanical systems. The former writes the mechanical deflection as $x(t) = Xe^{-i\omega t} + \text{c.c.}$, the latter as $x(t) = Xe^{i\omega t} + \text{c.c.}$ This alone suffices to arrive at different expressions for the mechanical susceptibility; rewriting them in our nomenclature, $\chi_\mathrm{m}(\omega) = \left[m(\omega_\mathrm{m}^2 - \omega^2 - i\omega\kappa_\mathrm{m})\right]^{-1}$ in Ref. 18, $\chi_\mathrm{m}(\omega) = \left[m(\omega_\mathrm{m}^2 - \omega^2 + i\omega\kappa_\mathrm{m})\right]^{-1}$ in Ref. 20. Note that no optical system or optomechanical coupling is required for the derivation of χ_m. The difference between the two expressions is in phase alone, leading to the same $|\chi_\mathrm{m}(\omega)|^2 = \left[m^2((\omega_\mathrm{m}^2 - \omega^2)^2 + \omega^2\kappa_\mathrm{m}^2)\right]^{-1}$. Furthermore, in the derivation of OMIT, χ_m is not inserted or directly used (since only optical observables are controlled and measured); at most, its expression evolves as part of the solution of the equations of motion.

The Fourier transformation of the measured microwave signal, corresponding to the choice of expression for frequency-dependent observables, is performed intrinsically by the vector network analyzer. This means that our experimental setup enforces a specific choice of sign convention when dealing with time-varying field amplitudes or charges. Does the choice matter? We can check this in a simple toy model. Let us assume a time-dependent input function $u(t)$, which undergoes Fourier transformation,

$$u(\omega) = \int e^{i\omega t} u(t)\,\mathrm{d}t \tag{13}$$

Now we assume a frequency-dependent transfer function $s(\omega)$ (corresponding to the transmission of our setup) is multiplied onto $u(\omega)$, and we obtain an output signal $v(t)$ via reverse Fourier transformation:

$$v(t) = \int e^{-i\omega t} s(\omega) u(\omega)\,\mathrm{d}\omega \tag{14}$$

In the above equations we have chosen one specific sign convention for the Fourier transformation; now we repeat the process with the opposite one:

$$u'(\omega) = \int e^{-i\omega t} u(t)\,\mathrm{d}t \tag{15}$$

$$v'(t) = \int e^{i\omega t} s'(\omega) u'(\omega)\,\mathrm{d}\omega \tag{16}$$

From Supplementary Equation 15 we see $u'(\omega) = u(-\omega)$. Inserting this into Supplementary Equation 16 and setting $v(t) = v'(t)$, we obtain as general relation between the two transfer functions

$$-s'(-\omega) = s(\omega) \tag{17}$$

Comparison with Supplementary Equation 10 tells us that the "wrong" sign convention leads to the negative, complex-conjugated value of A. However, as already noted above, the evaluated quantity in all our OMIT data sets is the absolute value squared transmission coefficient $|S_{21}|^2$ alone — and here, the difference between the two posssible approaches is lost. Since the choice of sign convention equally applies to the crosstalk channel, this conclusion remains valid for the extended version of Supplementary Equation 12, at most leading to different signs of the fit parameters r and θ.

The remaining question is whether the sign conventions of Ref. 18 are consistent between the different – "optical" and "mechanical" – subsystems of our microwave-optomechanical device. We can deliberately introduce a sign inconsistency into the ansatz of Weis *et al.*, Ref. 18, by replacing their Supplementary Equation S15 with

$$\delta x(t) = X e^{i\Omega t} + X^* e^{-i\Omega t} \tag{18}$$

It turns out that the derivation of Supplementary Equation 10 is equally possible this way; now the deflection amplitude X^* (instead of X) is eliminated from the equations, leading to the same result.

It remains to remark that the existence of any fundamental difference between "optical" and "microwave" optomechanics would be highly surprising and unexpected, given that the two types of experiment differ only in frequency range, and that a coplanar waveguide resonator can be treated using formalisms from both wave optics and lumped element electronic circuits.

Supplementary Note 11 — Modeling the geometric optomechanical coupling

Comparing our system with previous microwave optomechanical devices, see, e.g., Ref. 21, we can model the carbon nanotube as metallic and conductive, neglecting Coulomb blockade effects. The only part of the total cavity capacitance C_c which depends on the nanotube deflection is then the geometrical capacitance $C_\mathrm{g}(x)$ between gate finger and nanotube, thus

$$\frac{\partial C_\mathrm{c}}{\partial x} = \frac{\partial C_\mathrm{g}}{\partial x}. \tag{19}$$

We can approximate $C_\mathrm{g}(x)$ using the relation for a long conductive rod above an infinite conductive plane,

$$C_\mathrm{g}(x) = \frac{2\pi\varepsilon_0\varepsilon_\mathrm{r}\ell}{\cosh^{-1}\left(\frac{d-x}{r}\right)}, \tag{20}$$

where d is the distance between nanotube and gate at equilibrium, x is the deflection, r the nanotube radius, and ℓ the nanotube length. Our device has 250 nm vacuum and 200 nm cross-linked PMMA (with a typical dielectric constant of $\varepsilon_\mathrm{r}^\mathrm{PMMA} \simeq 3$)[22] between nanotube and gate; we approximate $d = 450$ nm and $\varepsilon_r = 2$. Since we do not know the diameter of our carbon nanotube, we assume a typical value of $r = 2$ nm.

From dc Coulomb blockade measurements, we obtain a gate capacitance $C_\mathrm{g}^\mathrm{CB} = 2.6$ aF; we identify this with the capacitance at zero deflection $C_\mathrm{g}(0)$, resulting in an *effective electronic length* of the system $\ell_\mathrm{eff} = 140$ nm, significantly smaller than the geometric length $\ell = 1000$ nm taken from the contact electrode distance. This indicates that our electrostatically induced potential well only takes up part of the nanotube length; the remaining parts represent barriers and/or leads.

From the abovementioned identification of $C_\mathrm{g}^\mathrm{CB} = C_\mathrm{g}(0)$ we obtain

$$\left.\frac{\partial C_\mathrm{g}}{\partial x}\right|_{x=0} = 0.95\,\frac{\mathrm{pF}}{\mathrm{m}} \tag{21}$$

and

$$g_0 = 2\pi \cdot 2.9\,\mathrm{mHz} \tag{22}$$

A summary of the device constants as input parameters and the predicted or measured optomechanical properties is given in Supplementary Table 1, where it is also compared with other optomechanical systems from literature.

			**meas. CNT CB enhanced**	Graphene drum (Singh *et al.*, 23)	Aluminum beam (Regal *et al.*, 21)
**"Optical" cavity**					
Resonance frequency		ω_c	$2\pi \cdot 5.74005\,\mathrm{GHz}$	$2\pi \cdot 5.9\,\mathrm{GHz}$	$2\pi \cdot 4.9\,\mathrm{GHz}$
Line width		κ_c	$2\pi \cdot 11.6\,\mathrm{MHz}$	$2\pi \cdot 242\,\mathrm{kHz}$	$2\pi \cdot 490\,\mathrm{kHz}$
Quality factor	ω_c/κ_c	Q_c	497	$[2.4 \cdot 10^4]$	10^4
Capacitance		C_c	875 fF	415 fF	
**Mechanical resonator**					
Resonance frequency		ω_m	$2\pi \cdot 502.5\,\mathrm{MHz}$	$2\pi \cdot 36\,\mathrm{MHz}$	$2\pi \cdot 240\,\mathrm{kHz}$
Line width		κ_m	$\sim 2\pi \cdot 50\,\mathrm{kHz}$	$2\pi \cdot 228\,\mathrm{Hz}$	$[2\pi \cdot 104\,\mathrm{Hz}]$
Quality factor	ω_m/κ_m	Q_m	$\sim 10^4$	$1.6 \cdot 10^5$	$2.3 \cdot 10^3$
Thermal occ. (10 mK)	$k_B T/\hbar\omega_m$	n_m	0.41	[5.8]	[870]
Length of nanotube / beam		ℓ	$1\,\mu\mathrm{m}$		$50\,\mu\mathrm{m}$
Effective electronic length		ℓ_{eff}	143 nm		
Radius of nanotube / drum / beam		r	2 nm	$4\,\mu\mathrm{m}$	$\sim 50\,\mathrm{nm}$
Effective mass		m	$4.8 \cdot 10^{-21}\,\mathrm{kg}$		$2 \cdot 10^{-15}\,\mathrm{kg}$
**Coupling**					
Zero-point motion ampl.	$\sqrt{\hbar/2m\omega_m}$	x_{zpf}	1.9 pm	29 fm	130 fm
Distance to gate / ground		d	450 nm	150 nm	$1\,\mu\mathrm{m}$
Dielectric constant		ε_r	2	1	1
Geometric capacitance to gate / ground		C_g	2.6 aF	578 aF	
Geometric gate coupling		$\partial C_g/\partial x$	0.95 pF/m		170 pF/m
**Optomechanical parameters**			(for optimal V_g)		
($n_{\mathrm{cav}} = 67500$)					
Side band resolution	ω_m/κ_c		43.5	149	0.5
Geometric single photon coupling		g_0	$2\pi \cdot 2.9\,\mathrm{mHz}$	$2\pi \cdot 0.83\,\mathrm{Hz}$	$[2\pi \cdot 0.15\,\mathrm{Hz}]$
Max. CB-enh. single photon coupling		g_0	$2\pi \cdot 95\,\mathrm{Hz}$		
Optomechanical coupling	$g_0\sqrt{n_{\mathrm{cav}}}$	g	$2\pi \cdot 24.7\,\mathrm{kHz}$	$[2\pi \cdot 216\,\mathrm{Hz}]$	$[2\pi \cdot 39\,\mathrm{Hz}]$
Cavity pull-in parameter	g_0/x_{zpf}	G	$2\pi \cdot 51\,\mathrm{Hz/pm}$	$2\pi \cdot 26.5\,\mathrm{Hz/pm}$	$2\pi \cdot 1.2\,\mathrm{Hz/pm}$
Dispersive coupling	g_0/κ_c	$\tilde{A}$	$8.2 \cdot 10^{-6}$	$[3.4 \cdot 10^{-6}]$	$[3.1 \cdot 10^{-7}]$
Maximal sideband cooling rate	$4n_{\mathrm{cav}}g_0^2/\kappa_c$	Γ_{opt}	$2\pi \cdot 211\,\mathrm{Hz}$	$[2\pi \cdot 0.77\,\mathrm{Hz}]$	$[2\pi \cdot 12\,\mathrm{mHz}]$
Cooperativity	$\Gamma_{\mathrm{opt}}/\kappa_m$	C	$4.2 \cdot 10^{-3}$	$[3.4 \cdot 10^{-3}]$	$[1.2 \cdot 10^{-4}]$
Cooling power	$\Gamma_{\mathrm{opt}}\hbar\omega_m$	$\dot{Q}$	$4.4 \cdot 10^{-22}\,\mathrm{W}$	$[1.2 \cdot 10^{-25}\,\mathrm{W}]$	$[1.2 \cdot 10^{-29}\,\mathrm{W}]$

Supplementary Table 1. **Overview of the parameters of the optomechanical system** The nomenclature of Ref. 16 is used. The geometric values for the carbon nanotube quantum dot are calculated based on the device geometry and dc measurement results; the Coulomb blockade enhanced values are based on the OMIT measurement evaluation, as are the derived parameters in the lowermost table block. Values of seminal early papers for a graphene drum, Ref. 23, and for an aluminum beam, Ref. 21, are given for comparison; parameters in brackets have been calculated based on the data in these publications.

Supplementary Note 12 — Coulomb-blockade enhancement of the optomechanical coupling

So far, the nanomechanical system has been approximated as metallic, and only geometric capacitances have been taken into account. We now demonstrate that the dispersive optomechanical coupling can be drastically enhanced when the quantum dot behavior of the nanotube and the resulting variation of its quantum capacitance is additionally considered. See also Refs. 17 and 24, where a nanotube is coupled *resonantly*, i.e., with $\omega_c \simeq \omega_m$, for a model based on similar principles, and Ref. 25 for the source of the derivation of the precise expression of the quantum capacitance.

We here assume that a full separation of time scales is possible, with $\omega_m \ll \omega_c \ll \Gamma$, where Γ characterizes the electronic tunnel processes of the quantum dot and corresponds to the lifetime broadening of its states; see Supplementary Note 3 for the validity of this assumption. The effective capacitance C_{eff} "visible" to the coplanar resonator is then given via the injected charge on the gate electrode $\langle Q_g \rangle$ averaged over single electron tunneling processes in the quantum dot at rates faster than both the mechanical frequency ω_m and the cavity frequency ω_c,

$$C_{\mathrm{eff}} = e\frac{\partial \langle Q_g \rangle}{\partial V_g}. \tag{23}$$

With V_{dot} as the potential of the quantum dot, $C_{\mathrm{dot}} = C_g + C_s + C_d$ as its total capacitance, and N as number of charge carriers on

it, electrostatics provides the relations $Q_\mathrm{g} = C_\mathrm{g}(V_\mathrm{g} - V_\mathrm{dot})$ and $V_\mathrm{dot} = (C_\mathrm{g}V_\mathrm{g} - eN)/C_\mathrm{dot}$, resulting in

$$\langle Q_\mathrm{g}\rangle = \frac{C_\mathrm{g}(C_\mathrm{s}+C_\mathrm{d})}{C_\mathrm{dot}} V_\mathrm{g} + e\langle N\rangle \frac{C_\mathrm{g}}{C_\mathrm{dot}} \tag{24}$$

The first term describes the geometric capacitance of the gate junction in series with source and drain junction; the second term stems from the quantum capacitance. The quantum capacitance thus becomes

$$C_\mathrm{CNT} = e\frac{C_\mathrm{g}}{C_\mathrm{dot}}\frac{\partial\langle N\rangle}{\partial V_\mathrm{g}}, \tag{25}$$

where $\langle N\rangle$ is the number of charge carriers on the quantum dot, averaged over fast single electron tunneling processes. Here, V_g is the static, externally applied gate voltage.

To calculate g_0, we require $\partial C_\mathrm{c}/\partial x$, and we now assume that this C_CNT (instead of C_g) is its dominant, deflection-dependent part, i.e.,

$$\frac{\partial C_\mathrm{c}}{\partial x} = \frac{\partial C_\mathrm{CNT}}{\partial x}. \tag{26}$$

Any change in the mechanically modulated geometric gate capacitance δC_g leads to a change of the gate voltage where Coulomb blockade oscillations reach maximum conductance; one can thus translate δC_g into an effective gate voltage modulation $\delta V_\mathrm{g}^\mathrm{eff}$ via

$$C_\mathrm{g}\delta V_\mathrm{g}^\mathrm{eff} = V_\mathrm{g}\delta C_\mathrm{g}, \tag{27}$$

where C_g is the static, geometric capacitance at zero deflection. With this we implicitly assume that a variation of the effective gate voltage $\delta V_\mathrm{g}^\mathrm{eff}$ acts on the system in the same way as a variation of the actual gate voltage δV_g. We now expand

$$\frac{\partial C_\mathrm{CNT}}{\partial x} = \frac{\partial C_\mathrm{CNT}}{\partial V_\mathrm{g}^\mathrm{eff}}\frac{\partial V_\mathrm{g}^\mathrm{eff}}{\partial x} = \frac{\partial C_\mathrm{CNT}}{\partial V_\mathrm{g}^\mathrm{eff}}\frac{V_\mathrm{g}}{C_\mathrm{g}}\frac{\partial C_\mathrm{g}}{\partial x} \tag{28}$$

Inserting Eq. (25) and using our definition of the effective gate voltage δV_g^eff, we can now simplify and obtain

$$\frac{\partial C_\mathrm{CNT}}{\partial x} = e\frac{\partial^2\langle N\rangle}{\partial V_\mathrm{g}^2}\frac{C_\mathrm{g}}{C_\mathrm{dot}}\frac{V_\mathrm{g}}{C_\mathrm{g}}\frac{\partial C_\mathrm{g}}{\partial x}. \tag{29}$$

In an intuitive picture, the cavity probes the *first* derivative of the charge $\langle N\rangle$; an optomechanical interaction results precisely when that derivative changes over a mechanical modulation cycle, i.e, when the *second* derivative of $\langle N\rangle$ is non-zero.

What remains is to insert an expression for $\langle N\rangle(V_\mathrm{g})$. Given our above requirements of large single electron tunneling rates, the limit $\hbar\Gamma \gg k_\mathrm{B}T$ of a lifetime-broadened single electron tunneling (SET) conductance peak at zero temperature is appropriate. Then, the time-averaged number of charge carriers on the quantum dot $\langle N\rangle(V_\mathrm{g})$, near a SET peak centered at V_g0, can be modelled as the integral over the quantum dot density of states below the Fermi energy,

$$\langle N\rangle(V_\mathrm{g}) = N_0 + \int_{-\infty}^{V_\mathrm{g}} \frac{1}{2\pi}\frac{\hbar\Gamma}{\left(\frac{\hbar\Gamma}{2}\right)^2 + e\alpha\left(V_\mathrm{g}' - V_\mathrm{g0}\right)^2}\,\mathrm{d}V_\mathrm{g}' \tag{30}$$

Here, N_0 is an offset number of charge carriers, and α is the gate lever arm.

In the fit of Figure 3 of the main text, the free parameters were the broadening Γ, the Coulomb oscillation position V_g0, and an additionally introduced scaling prefactor a, resulting in $V_\mathrm{g0} = -1.18841\,\mathrm{V}$, $\hbar\Gamma = 0.673\,\mathrm{meV}$, and $a = 5.77 = g_0^\mathrm{exp}/g_0^\mathrm{th}$.

The broadening Γ corresponds to a frequency of 163 GHz, clearly consistent with the separation of time scales. Note that in the case of two unequal tunnel barriers defining the quantum dot, the current through the nanotube is limited by the more opaque barrier, while the broadening is dominated by the more transparent barrier. Thus, the tunnel current only provides a lower boundary for Γ.

Supplementary Note 13 — Consistency with conductance

For the OMIT measurements, to reach a sharp Coulomb oscillation and with that a strong optomechanical enhancement effect, we have tuned the bias voltage to zero as precisely as possible. An ac conductance measurement is challenging at the high device

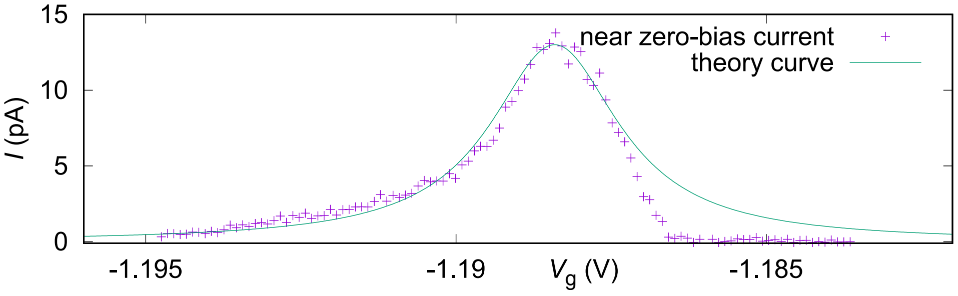

Supplementary Figure 12. **Comparison of the predicted conductance from Figure 3b of the main manuscript and the recorded current** Data points, current due to bias offsets in the measurement and rectification effects, recorded in the course of a red sideband upconversion experiment similar to Figure 4 in the main manuscript. Solid line, fit result curve of the conductance from Figure 3b. The position in V_g as well as the scaling in I have been adapted, however, the curve widths are unscaled.

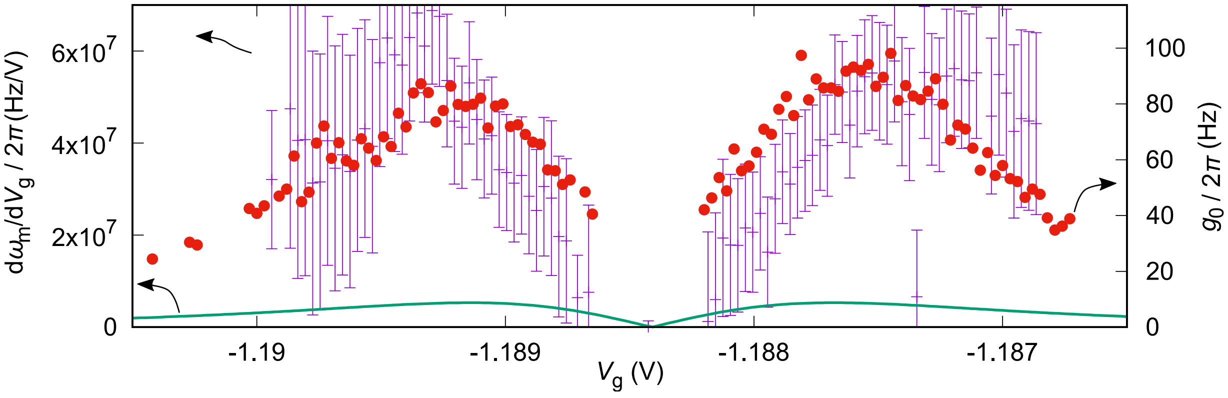

Supplementary Figure 13. **Comparison of vibration frequency softening and optomechanical coupling** Solid red points, optomechanical single photon coupling $g_0(V_g)$ in Hz, as also plotted in Figure 2g, right axis; bars, numerical derivative of the extracted mechanical resonance frequency, $d\omega_m/dV_g\ /2\pi$ in Hz/V, left axis; green solid line, expected $d\omega_m/dV_g\ /2\pi$ from the electrostatic model, left axis, see also the text. The error bars are derived from the standard error of the mechanical resonance frequency in the OMIT fit, cf. Figure 2f.

impedance; while the dc current through the device can be measured, it is typically dominated by minute voltage offsets and rectification effects.[26]

Supplementary Figure 12 compares such a current trace, extracted from a separate data set and recorded at nominally zero bias, with the theoretical conductance curve obtained from the fit in Figure 3b of the main text. An offset in V_g has been corrected, and the curve has been scaled in height (I) to coincide, however, the width of both peaks is unscaled.

The asymmetry of the current peak can be attributed to abovementioned rectification effects; nevertheless, the width of the conductance curve derived from the OMIT data and of the current peak coincide nicely.

Supplementary Note 14 — Consistency with frequency softening

Figure 2f of the main text not only demonstrates the optomechanical coupling, but also allows us to trace the mechanical resonance frequency and its electrostatic softening at the position of the charge degeneracy point[7]. The change in effective spring constant is given by[7]

$$\Delta k = \frac{V_g(V_g - V_{dot})}{C_{dot}} \left(\frac{\partial C_g}{\partial x}\right)^2 \left(1 - |e| \frac{\partial \langle N \rangle}{\partial (C_g V_g)}\right). \tag{31}$$

Here, C_{dot} is the total quantum dot capacitance and V_{dot} is the potential of the quantum dot.

In the case of $V_{sd} = 0$, $V_{dot}(V_g)$ oscillates around the common potential of source and drain contact. We can approximate $V_{dot} = 0$, an assumption that becomes better with increasing $|V_g|$. Neglecting now constant terms and assuming that $\langle N \rangle$ is only

a function of V_g, the change in spring constant reduces to

$$\Delta k = -|e| \frac{V_g^2}{C_{dot} C_g} \left(\frac{\partial C_g}{\partial x} \right)^2 \frac{\partial \langle N \rangle}{\partial V_g}. \tag{32}$$

Comparing Supplementary Equations 29 and 32, we see that the derivative of the mechanical resonance frequency $\partial \omega_m / \partial V_g$ should be proportional to the optomechanical coupling. We obtain for the gate voltage derivative of the mechanical resonance frequency the at the Coulomb oscillation dominant term

$$\frac{d\omega_m}{dV_g} = \frac{1}{2m\omega} \frac{d(\Delta k)}{dV_g} \approx \frac{e}{2m\omega} \frac{V_g^2}{C_{dot} C_g} \left(\frac{\partial C_g}{\partial x} \right)^2 \frac{\partial^2 \langle N \rangle}{\partial V_g^2} \tag{33}$$

This correspondence is illustrated in Supplementary Figure **13**. Even after applying a sliding average, the numerical derivative $\partial \omega_m / \partial V_g$ remains noisy. The qualitative evolution agrees approximately with the extracted optomechanical coupling.

The green solid line in Supplementary Figure **13** results from inserting the values of our electrostatic and mechnanical model into Supplementary Equation 33. The experimentally observed softening exceeds the model results by approximately a factor 10. The origin of this deviation is so far unknown; with the deflection derivative of the gate capacitance, the mass, and the total quantum dot capacitance however several parameters enter also here that are experimentally not precisely known, see the following Supplementary Note **15**.

Supplementary Note 15 — Error sources

In the following we discuss potential errors introduced by approximations entering our calculation.

- The length of the vibrating nanotube segment is assumed to be equal to the contact electrode separation, $\ell = 1\,\mu$m. It can be larger in case the nanotube is not aligned perpendicular to the electrodes.
- Since we do not know the radius of the specific carbon nanotube in the device, we assume a typical value $r = 2$ nm.
- The space between nanotube and gate electrode consists of 250 nm vacuum and 200 nm cross-linked PMMA. We simplify this as a single material with relative dielectric constant $\varepsilon_r = 2$ and thickness $d = 450$ nm.
- We approximate the nanotube-gate capacitance as that of a thin, long rod above an infinite conductive plane, while the width of the gate electrode, below the nanotube and perpedicular to it, is also only approximately 100 nm.
- The effective mass of the carbon nanotube vibrational resonator is assumed to be half its total mass, $m = \left(2\pi r \ell \rho_{graphene}\right)/2$.

Supplementary Note 16 — Additional data

Supplementary Figure **14** displays a red sideband upconversion measurement analogous to Figure 4 of the main text, but recorded at the subsequent Coulomb blockade oscillation. The plot exhibits essentially the same behaviour, with the frequency of the upconversion feature following the electrostatic softening at the charge degeneracy point[7] and a finite coupling. Away from the charge degeneracy point, no signal is found, indicating a negligible optomechanical coupling in agreement with our findings.

---

[1] S. Blien, P. Steger, A. Albang, N. Paradiso, and A. K. Hüttel, "Quartz tuning-fork based carbon nanotube transfer into quantum device geometries," Phys. Stat. Sol. B **255**, 1800118 (2018).

[2] D. Yuan, L. Ding, H. Chu, Y. Feng, T. P. McNicholas, and J. Liu, "Horizontally aligned single-walled carbon nanotube on quartz from a large variety of metal catalysts," Nano Letters **8**, 2576–2579 (2008).

[3] M. Kumar and Y. Ando, "Chemical vapor deposition of carbon nanotubes: a review on growth mechanism and mass production," Journal of Nanoscience and Nanotechnology **10**, 3739–3758 (2010).

[4] Y. V. Nazarov and Y. M. Blanter, *Quantum transport: Introduction to Nanoscience* (Cambridge Univ. Press, Cambridge, 2009).

[5] S. Reinhardt, L. Pirker, C. Bäuml, M. Remškar, and A. K. Hüttel, "Coulomb blockade spectroscopy of a MoS_2 nanotube," physica status solidi (RRL) – Rapid Research Letters **13**, 1900251 (2019).

[6] A. K. Hüttel, G. A. Steele, B. Witkamp, M. Poot, L. P. Kouwenhoven, and H. S. J. van der Zant, "Carbon nanotubes as ultrahigh quality factor mechanical resonators," Nano Letters **9**, 2547–2552 (2009).

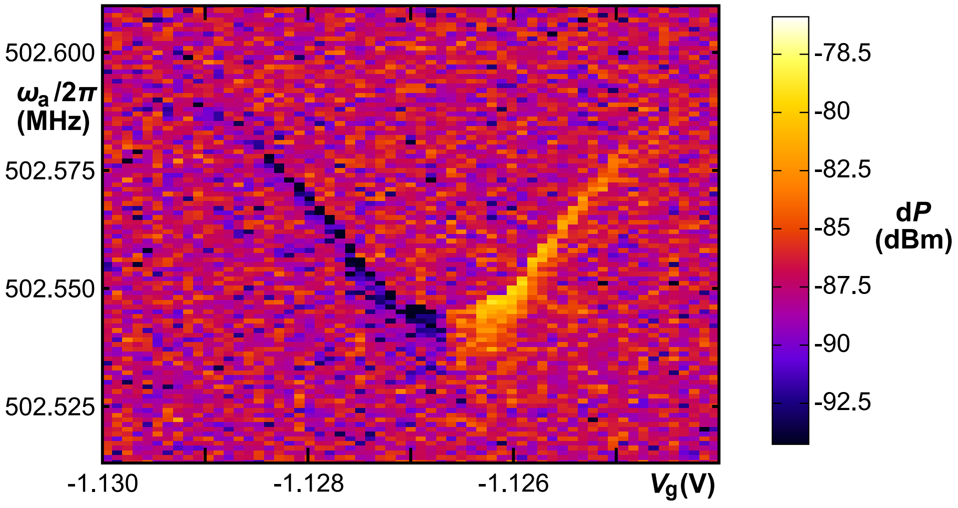

Supplementary Figure 14. **Subsequent Coulomb oscillation** Red sideband upconversion experiment analogous to Figure 4 of the main text, but performed at the subsequent Coulomb oscillation.

[7] G. A. Steele, A. K. Hüttel, B. Witkamp, M. Poot, H. B. Meerwaldt, L. P. Kouwenhoven, and H. S. J. van der Zant, "Strong coupling between single-electron tunneling and nanomechanical motion," Science **325**, 1103–1107 (2009).

[8] D. R. Schmid, P. L. Stiller, C. Strunk, and A. K. Hüttel, "Magnetic damping of a carbon nanotube nano-electromechanical resonator," New J. Phys. **14**, 083024 (2012).

[9] K. J. G. Götz, D. R. Schmid, F. J. Schupp, P. L. Stiller, Ch. Strunk, and A. K. Hüttel, "Nanomechanical characterization of the Kondo charge dynamics in a carbon nanotube," Phys. Rev. Lett. **120**, 246802 (2018).

[10] V. Sazonova, Y. Yaish, H. Üstünel, D. Roundy, T. A. Arias, and P. L. McEuen, "A tunable carbon nanotube electromechanical oscillator," Nature **431**, 284–287 (2004).

[11] C. C. Wu and Z. Zhong, "Capacitive spring softening in single-walled carbon nanotube nanoelectromechanical resonators," Nano Letters **11**, 1448–1451 (2011).

[12] P. L. Stiller, S. Kugler, D. R. Schmid, C. Strunk, and A. K. Hüttel, "Negative frequency tuning of a carbon nanotube nano-electromechanical resonator under tension," Phys. Stat. Sol. B **250**, 2518–2522 (2013).

[13] P. J. Petersan and S. M. Anlage, "Measurement of resonant frequency and quality factor of microwave resonators: Comparison of methods," Journal of Applied Physics **84**, 3392–3402 (1998).

[14] R. N. Simons, *Coplanar Waveguide Circuits, Components, and Systems* (John Wiley & Sons, Inc., 2001).

[15] M. Göppl, A. Fragner, M. Baur, R. Bianchetti, S. Filipp, J. M. Fink, P. J. Leek, G. Puebla, L. Steffen, and A. Wallraff, "Coplanar waveguide resonators for circuit quantum electrodynamics," Journal of Applied Physics **104**, 113904 (2008).

[16] M. Aspelmeyer, T. J. Kippenberg, and F. Marquardt, "Cavity optomechanics," Rev. Mod. Phys. **86**, 1391–1452 (2014).

[17] N. Ares, T. Pei, A. Mavalankar, M. Mergenthaler, J. H. Warner, G. A. D. Briggs, and E. A. Laird, "Resonant optomechanics with a vibrating carbon nanotube and a radio-frequency cavity," Phys. Rev. Lett. **117**, 170801 (2016).

[18] S. Weis, R. Rivière, S. Deléglise, E. Gavartin, O. Arcizet, A. Schliesser, and T. J. Kippenberg, "Optomechanically induced transparency," Science **330**, 1520–1523 (2010).

[19] O. Shevchuk, V. Singh, G. A. Steele, and Y. M. Blanter, "Optomechanical response of a nonlinear mechanical resonator," Phys. Rev. B **92**, 195415 (2015).

[20] K. Lehnert, "Introduction to microwave cavity optomechanics," in *Cavity Optomechanics*, edited by M. Aspelmeyer, T. J. Kippenberg, and F. Marquardt (Springer-Verlag Berlin Heidelberg, 2014) Chap. 11.

[21] C. A. Regal, J. D. Teufel, and K. W. Lehnert, "Measuring nanomechanical motion with a microwave cavity interferometer," Nature Physics **4**, 555–560 (2008).

[22] J. A. Brydson, *Plastics Materials*, 7th ed. (Butterworth-Heinemann, 1999).

[23] V. Singh, S. J. Bosman, B. H. Schneider, Y. M. Blanter, A. Castellanos-Gomez, and G. A. Steele, "Optomechanical coupling between a multilayer graphene mechanical resonator and a superconducting microwave cavity," Nature Nanotechnology **9**, 820–824 (2014).

[24] Y. Wen, N. Ares, T. Pei, G. A. D. Briggs, and E. A. Laird, "Measuring carbon nanotube vibrations using a single-electron transistor as a fast linear amplifier," Applied Physics Letters **113**, 153101 (2018).

[25] T. Duty, G. Johansson, K. Bladh, D. Gunnarsson, C. Wilson, and P. Delsing, "Observation of quantum capacitance in the Cooper-pair transistor," Phys. Rev. Lett. **95**, 206807 (2005).

[26] J. Weis, R. J. Haug, K. von Klitzing, and K. Ploog, "Single-electron tunnelling transistor as a current rectifier with potential-controlled current polarity," Semiconductor Science and Technology **10**, 877–880 (1995).
